# Novel body component score predicts long‐term survival in patients with stage I–III colorectal cancer following radical resection

**DOI:** 10.1002/ags3.12890

**Published:** 2024-11-26

**Authors:** Takashi Aida, Teppei Kamada, Taigo Hata, Junji Takahashi, Eisaku Ito, Kenei Furukawa, Masashi Yoshida, Hironori Ohdaira, Toru Ikegami, Yutaka Suzuki

**Affiliations:** ^1^ Department of Surgery International University of Health and Welfare Hospital Tochigi Japan; ^2^ Department of Surgery The Jikei University School of Medicine Tokyo Japan

**Keywords:** body component, colorectal cancer, ectopic fat, skeletal muscle, somatic fat

## Abstract

**Background:**

In gastrointestinal cancer, the relationship among skeletal muscle, subcutaneous and visceral fat mass, and prognosis is gaining attention. Herein, we developed a body component score (BCS) to comprehensively evaluate total body composition in patients with stage I–III colorectal cancer (CRC) and examined its relationship with long‐term prognosis.

**Methods:**

This retrospective study included 300 patients with CRC who underwent curative colorectal resection in 2010–2019. The BCS included skeletal muscle index (SMI), subcutaneous fat area (SFA), visceral fat area (VFA), fatty liver, and pancreatic fatty replacement, measured by preoperative computed tomography. The BCS was calculated as the sum of each score from 0 to 5; patients were grouped into low (score 0–1), medium (score 2–3), and high (score 4–5) BCS. Multivariate Cox proportional hazard models assessed disease‐free (DFS) and cancer‐specific survival (CSS) in these patients.

**Results:**

Multivariate analysis showed that T3 or T4 tumors (*p* = 0.038), pathological stage III (*p* < 0.001), and low BCS [*p* = 0.016; hazard ratio (HR), 1.95; 95% confidence interval (CI), 1.13–3.35] were independently associated with DFS, whereas pathological stage III (*p* < 0.001) and low BCS (*p* = 0.001; HR, 3.14; 95% CI, 1.57–6.27) were independent prognostic factors for CSS. Patients with a low BCS had significantly worse DFS (*p* < 0.001) and CSS (*p* < 0.001), according to the log‐rank test for trends.

**Conclusions:**

The BCS may effectively predict prognosis in patients with CRC.

## Graphical abstract

The body component score (BCS) consists of three key elements: skeletal muscle, bodily fat, and ectopic fat. BCS can comprehensively represent the states of body composition and predict the long‐term survival of resectable colorectal cancer following radical resection.

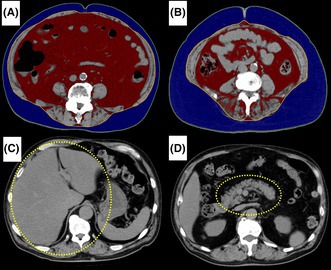

## 1 INTRODUCTION

Colorectal cancer (CRC) is the second leading cause of cancer‐related deaths and the third most common malignancy.^1^ Identifying and assessing prognostic factors for CRC are crucial for improving treatment outcomes of this malignancy. Recently, evaluating body composition in patients with cancer has gained attention because of the association between reduced skeletal muscle and visceral and subcutaneous adipose tissue with survival in cancers, including CRC.^2^, ^3^ Ectopic fat accumulation, such as in the fatty liver, may influence CRC survival and improve prognosis for non‐metastatic CRC.^4^ Although the relationship between each body component and CRC prognosis has been studied, a comprehensive index including skeletal muscle and subcutaneous, visceral, and ectopic fat does not exist currently. The prognostic impact of interactions between these body components remains unclear. Therefore, this retrospective study aimed to ascertain the combined impact of skeletal muscle, body fat, and ectopic fat on long‐term CRC outcomes. We developed a novel total body component score (BCS) and investigated its prognostic significance in patients with stage I–III CRC following curative resection.

## 2 MATERIALS AND METHODS

### 2.1 Patient selection

This retrospective cohort study included 300 patients with CRC who underwent radical resection at the Department of Surgery, International University of Health and Welfare Hospital, Tochigi, Japan, between January 2011 and March 2020. Patients with distant metastases (stage IV), other malignancies, postoperative mortality, or unavailable body component data were excluded. Data regarding clinical information, operative and pathological findings, and the postoperative course were collected from medical records. Patients were enrolled until cancer‐specific death or others or the end of follow‐up. This study was conducted in accordance with the Declaration of Helsinki and approved by the Institutional Review Board of the International University of Health and Welfare Hospital (approval no. 21‐B‐22). All data were subject to strict privacy policies, and patients and their family members could withdraw from the study at any time. The requirement for informed consent was waived because of the retrospective design of the study.

### 2.2 Treatment and follow‐up

The type of resection was determined based on preoperative tumor staging and tumor‐node‐metastasis (TNM) classification for CRC staging, referring to tumor pathology and the *General Rules for the Clinical and Pathological Study of Primary Colorectal Cancer by the Japanese Classification of Colorectal*, *Appendiceal*, *and Anal Carcinoma* (3rd English Edition).^5^

Surgical resection was performed laparoscopically and classified as ileocecal resection, right or left hemicolectomy, transverse colectomy, sigmoid colectomy, anterior rectal resection, Hartmann's procedure, abdominoperineal resection, and intersphincteric resection, following the Japan Society for Cancer of the Colon and Rectum guidelines 2019.^6^ Right‐sided CRC was defined tumors in the cecum, ascending colon, and middle transverse colon; left‐sided CRC was defined as tumors near the splenic flexure and beyond.^6^ Comorbidities were evaluated using the Charlson Comorbidity Index (CCI).^7^ Basic post‐primary surgery surveillance included tumor marker monitoring using serum CEA and CA19‐9 levels, enhanced CT evaluations, and colonoscopy. Tumor markers CEA and CA19‐9 were routinely examined every 3 months, enhanced CT of the chest and abdomen every 6 months, and colonoscopies every 1–2 years.^8^

Recurrence was defined as new detected local or distant metastatic tumors using enhanced CT, magnetic resonance imaging, or positron emission tomography with CT, regardless of increased tumor marker levels.

### 2.3 Date collection

Biochemical blood tests were performed in all patients within 1 week preoperatively. Clinicopathological variables included age, sex, body mass index, tumor location, preoperative obstruction caused by the tumor, TNM stage, histopathological type, pathological findings (residual tumor), surgical procedure, postoperative infectious complications (anastomotic leakage or surgical site infection or ileus), CCI, adjuvant chemotherapy, operative time, intraoperative blood loss, preoperative serum albumin level, C‐reactive protein level, CEA level, and CA19‐9 level. The staging of T and N factors was determined by pathological findings.

### 2.4 Definition of body component score

**FIGURE 1:**
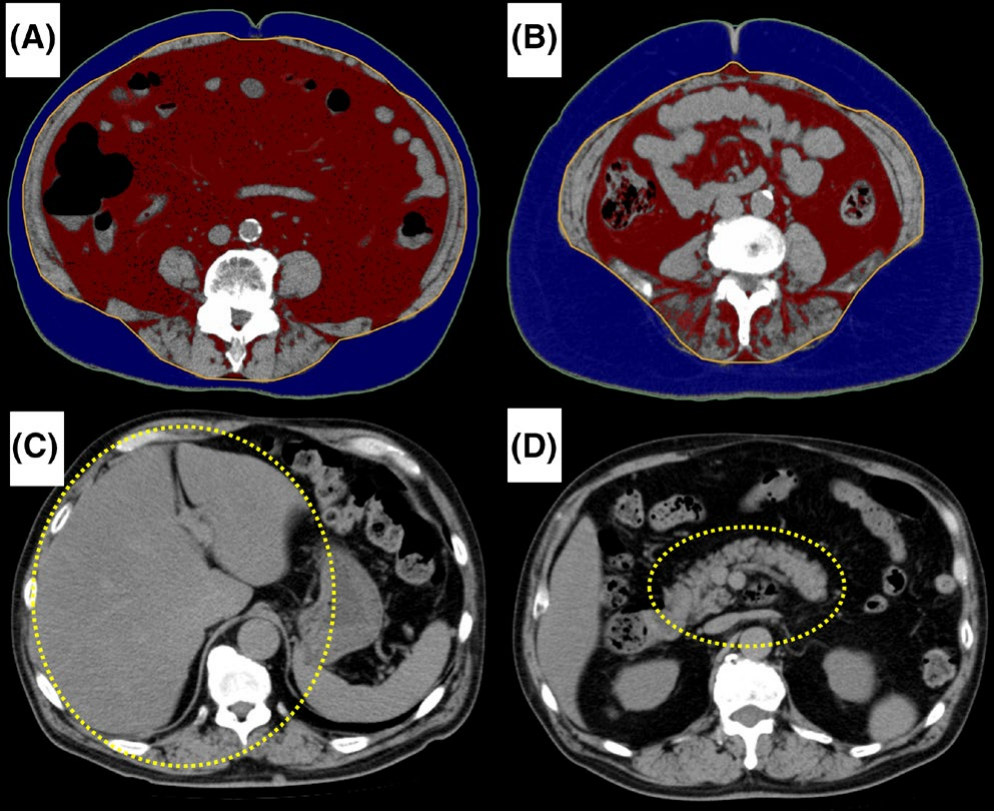
Analysis of visceral fat areas, subcutaneous fat areas, fatty liver, and pancreatic fatty replacement. Computed tomography (CT) imaging analysis at the umbilicus level in the axial plane: Red areas indicate visceral fat areas (VFAs), whereas blue areas indicate subcutaneous fat areas (SFAs). The presence of fatty liver and pancreatic fatty replacement was verified via abdominal computed tomography (CT) images, highlighted in yellow circles. (A) Patient with high VFA. (B) Patient with high SFA. (C) Patient with a fatty liver. (D) Pancreatic fatty replacement.

The skeletal muscle index (SMI), visceral fat area (VFA), subcutaneous fat area (SFA), and ectopic fat were measured using CT within 1 month preoperatively. The SMI was defined as the median area of the psoas muscle mass (length of the major axis multiplied by length of the minor axis) at the third lumbar vertebra, divided by the square of the height (cm^2^/m^2^).^9^ VFA and SFA were measured at the umbilicus level using an image analysis system (Ziosoft1 Inc., Tokyo, Japan; Figure 1A,B).^10^ Ectopic fat was concomitantly evaluated with or without fatty liver and pancreatic fatty replacement (Figure 1C,D). We define fatty liver as a threshold of ≤48 Hounsfield units (HU) and fatty pancreas at ≤36 HU, using a standard region of interest technique in abdominal CT.^11^, ^12^, ^13^ Cut‐off values for SMI, VFA, SFA were determined by receiver operating characteristics analysis using 5‐year survival status based on the Youden Index (the maximum points of “sensitivity + specificity—1”) for each sex. High SMI, VFA, or SFA was scored as 1, whereas low values were scored as 0. The presence of fatty liver or pancreatic fatty replacement was scored as 1, whereas their absence was scored as 0.

The BCS was calculated as the sum of scores from 0 to 5 points. Patients were classified into three groups based on their BCS: low BCS (score 0–1), medium BCS (score 2–3), and high BCS (score 4–5).

### 2.5 Statistical analyses

All statistical analyses were performed using EZR, version 1.54. Statistical significance was set at *p* < 0.05. Clinicopathological data are expressed as medians, interquartile ranges (IQRs), or ratios. Continuous and categorical variables were compared using the Mann–Whitney *U* test or chi‐square test, as appropriate. Univariate and multivariate Cox proportional hazards regression models were used to estimate hazard ratios (HR) for disease‐free survival (DFS) and cancer‐specific survival (CSS). The multivariate Cox regression model included significant factors without multicollinearity from the univariate analysis. Cumulative survival probabilities were estimated using the Kaplan–Meier method, and linear trends in survival probabilities across groups were assessed using the log‐rank test.

## 3 RESULTS

### 3.1 Patient clinicopathological characteristics

Table 1 presents the clinicopathological characteristics of patients, shown as medians, IQRs, or ratios. A low BCS was observed in 93 patients (31%), medium BCS in 120 (40%), and high BCS in 87 (29%). Figure S1 displays the proportions of each parameter (A–E) in the three BCS groups. The median age of the cohort was 72 years, including 186 men and 114 women. The median follow‐up periods for determining DFS and CSS were 4.0 (IQR, 2.2–5.5 years) and 4.4 years (IQR, 2.7–5.5 years), respectively. During the follow‐up period, 56 of 300 patients experienced tumor recurrence (19%) and 52 died (17%). The cancer‐specific mortality occurred in 43 patients with CRC (14%). Patients with a low BCS were older (*p* = 0.003) and had a significantly lower body mass index (*p* < 0.001) than those with a medium or high BCS. Significantly more N2 or N3 lymph node metastases (*p* = 0.017) were observed in patients with a low BCS than in those with a medium or high BCS; however, no significant difference was observed in the pathological stage (*p* = 0.421). There were also no significant differences in the R0/1/2 resection rates (*p* = 0.681) or in adjuvant chemotherapy (*p* = 0.447). A total of 124 patients (41%) received adjuvant chemotherapy, with 48 treated with an Oxaliplatin‐based regimen (i.e. FOLFOX, XELOX), 65 with Tegafur‐uracil plus calcium folinate, and 11 with other regimens (i.e. TS‐1). Serum albumin levels (*p* = 0.006) were significantly lower and serum CEA levels were significantly higher in patients with a low BCS than in those with a medium or high BCS.

**TABLE 1:** Clinicopathological characteristics of enrolled patients and univariate analysis of clinicopathologic variables in relation to the Body component score.

		Body component score		
Variables	Total (*n* = 300)	Low (*n* = 93)	Med (*n* = 120) or high (*n* = 87)	*p*‐value
Age, (years)	72 (65–79)	75 (68–81)	71 (63–78)	0.003
Sex, male	186 (62%)	51 (55%)	135 (65%)	0.096
Body mass index, (kg/m^2^)	22 (20–25)	20 (18–22)	23 (21–26)	<0.001
Tumor location, right‐sided colon cancer	88 (29%)	31 (30%)	57 (28%)	0.338
Obstructive colorectal cancer	22 (7%)	10 (11%)	12 (6%)	0.152
Histological type				
tub1	143 (48%)	47 (51%)	96 (46%)	0.333
tub2	145 (48%)	42 (45%)	103 (50%)	
others	12 (4%)	4 (4%)	8 (4%)	
T factor				
T1	61 (20%)	13 (14%)	48 (24%)	0.215
T2	48 (16%)	15 (16%)	33 (16%)	
T3	170 (57%)	56 (60%)	114 (55%)	
T4	21 (7%)	9 (10%)	12 (6%)	
N factor				
N0	185 (62%)	55 (59%)	130 (63%)	0.017
N1	74 (24%)	18 (19%)	56 (27%)	
N2	36 (12%)	16 (17%)	20 (10%)	
N3	5 (2%)	4 (4%)	1 (0.5%)	
Pathological stage				
I	90 (30%)	23 (25%)	67 (32%)	0.421
II	95 (32%)	32 (34%)	63 (30%)	
III	115 (38%)	38 (41%)	77 (37%)	
Adjuvant chemotherapy, yes	124 (41%)	35 (38%)	89 (43%)	0.447
Oxaliplatin based	48 (16%)	15 (16%)	33 (16%)	
Tegafur‐uracil + calcium folinate	65 (22%)	16 (17%)	49 (24%)	
Others	11 (4%)	4 (4%)	7 (3%)	
Oxaliplatin based regimen completion rate	40 / 300 (13%)	12 / 93 (13%)	28 / 207 (14%)	0.692
Operative procedure				
Ileocecal resection	40 (13%)	16 (17%)	24 (12%)	0.509
Right hemicolectomy	44 (15%)	14 (15%)	30 (14%)	
Transverse colectomy	3 (1%)	1 (1%)	2 (1%)	
Left hemicolectomy	17 (6%)	4 (4%)	13 (6%)	
Sigmoid colectomy or High anterior resection	95 (32%)	25 (27%)	70 (34%)	
Low anterior resection or Hartmann's procedure	67 (22%)	19 (20%)	47 (23%)	
Abdominoperineal resection	34 (11%)	13 (14%)	21 (10%)	
Lymph node dissection				
D1	12 (4%)	5 (5%)	7 (3%)	0.583
D2	115 (38%)	33 (35%)	82 (39%)	
D3	173 (58%)	55 (59%)	118 (57%)	
Residual tumor				
R0	293 (98%)	90 (97%)	203 (98%)	0.681
R1	7 (2%)	3 (3%)	4 (2%)	
R2	0 (0%)	0 (0%)	0 (0%)	
Operative time (min)	263 (210–323)	246 (195–312)	264 (217–325)	0.072
Intraoperative blood loss (mL)	20 (5–60)	15 (5–50)	20 (5–60)	0.672
Postoperative complication	93 (31%)	32 (34%)	61 (29%)	0.419
Anastomotic leakage	13 (4%)	4 (4%)	9 (4%)	1.000
Surgical site infection	46 (15%)	16 (17%)	30 (14%)	0.604
Ileus	37 (12%)	12 (13%)	25 (12%)	0.851
Charlson Comorbidity Index				
Medium (1, 2)	183 (61%)	62 (67%)	121 (59%)	0.371
High (3, 4)	101 (34%)	26 (28%)	75 (36%)	
Very high (≥5)	16 (5%)	5 (5%)	11 (5%)	
Serum CRP level (mg/dL)	0.14 (0.06–0.36)	0.10 (0.07–0.35)	0.15 (0.04–0.46)	0.202
Serum albumin level (g/dL)	4.0 (3.6–4.2)	3.7 (3.4–4.1)	4.0 (3.7–4.3)	<0.001
Serum CEA level (ng/mL)	3.4 (2.0–6.3)	4.7 (2.3–9.4)	3.2 (1.9–5.3)	0.001
Serum CA19‐9 level (U/mL)	9.4 (4.4–21)	10 (4.6–33)	8.9 (4.2–19)	0.113

Abbreviations: CA19‐9, carbohydrate antigen 19–9; CEA, carcinoembryonic antigen; CRP, C‐reactive protein; tub1 (2), well (moderately)‐differentiated tubular adenocarcinoma.

### 3.2 Univariate and multivariate analyses of prognostic factors for disease‐free and cancer‐specific survival in patients with stage I–III CRC

**FIGURE 2:**
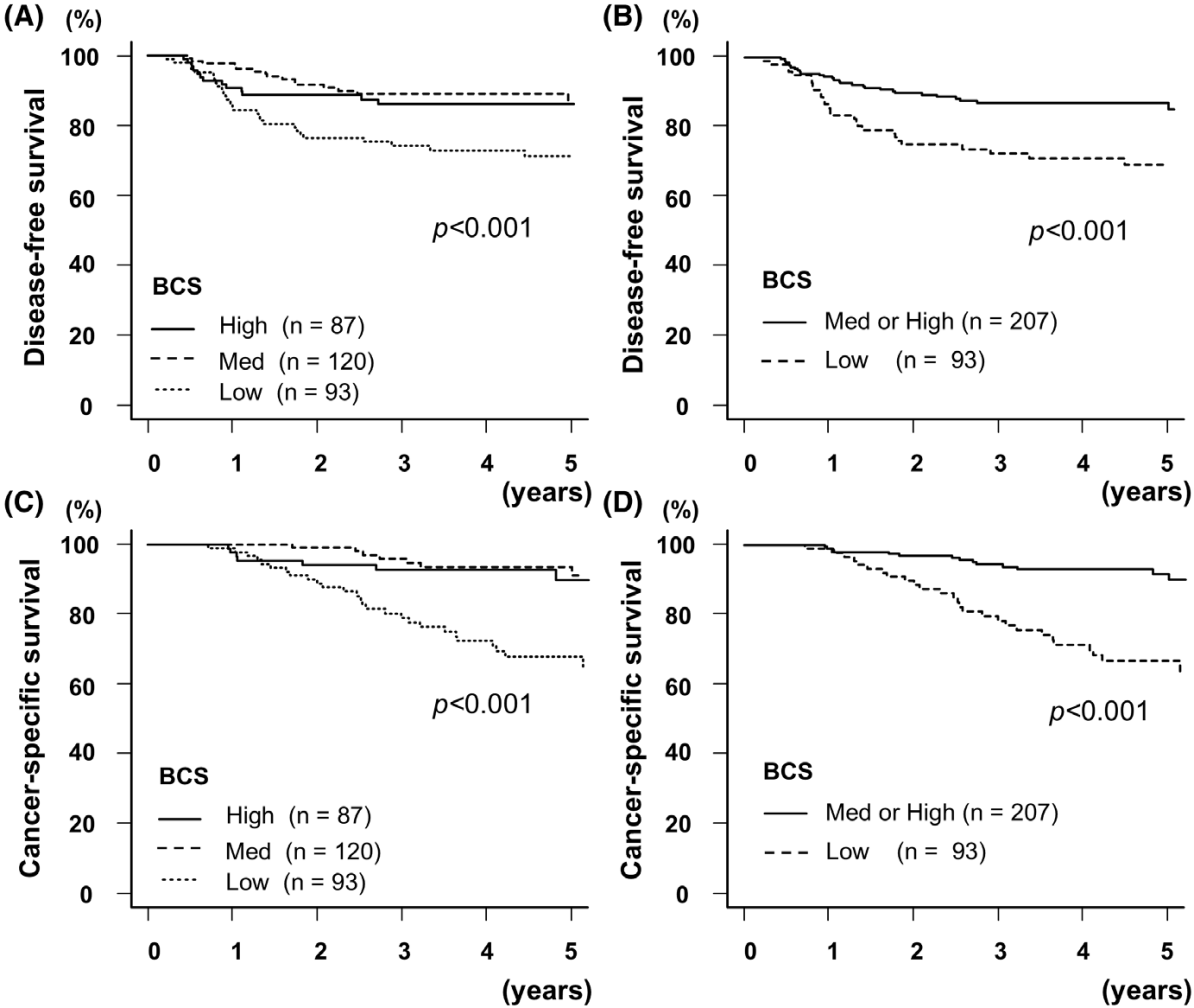
Kaplan–Meier curves according to the status of each body component score (BCS) group. Kaplan–Meier curves of disease‐free (A, B) and cancer‐specific survival (C, D) according to the BCS status after resection for stage I–II CRC.

Table 2 displays the associations between clinical variables and DFS following resection for stage I–III CRC. Univariate analysis revealed significant associations with DFS for T3 or T4 tumors (*p* < 0.001), adjuvant chemotherapy (*p* < 0.001), R1 resection (*p* = 0.006), pathological stage III (*p* < 0.001), serum CEA level ≥5.0 ng/mL (*p* < 0.001), serum CA19‐9 level ≥ 37.0 U/mL (*p* < 0.001), low VFA (*p* = 0.005), low SMI (*p* = 0.006), and low BCS (*p* = 0.002). Multivariate analysis identified T3 or T4 tumors (*p* = 0.038; HR 2.61; 95% CI, 1.06–6.43), pathological stage III (*p* < 0.001; HR, 4.25; 95% CI, 1.94–9.30), and low BCS (*p* = 0.016; HR, 1.95; 95% CI, 1.13–3.35) as independent predictors of DFS. A low BCS was significantly associated with worse DFS (*p* < 0.001; log‐rank test for trend) (Figure 2A,B).

**TABLE 2:** Univariate and multivariate analyses of prognostic factors for disease‐free survival in patients with colorectal cancer after resection.

	Univariate analysis		Multivariate analysis	
Variables	HR (95% CI)	*p‐*value	HR (95% CI)	*p*‐value
Age ≥ 75 years	1.01 (0.59–1.73)	0.976		
Sex, male	1.37 (0.78–2.40)	0.272		
Body mass index <22 kg/m^2^	1.45 (0.86–2.44)	0.169		
Right‐sided colon cancer, yes	0.95 (0.53–1.70)	0.867		
T3 or T4 tumor, yes	5.54 (2.38–12.9)	<0.001	2.61 (1.06–6.43)	0.038
Adjuvant chemotherapy, yes	3.45 (1.97–6.16)	<0.001	0.96 (0.46–1.98)	0.918
R1 resection, yes	4.16 (1.50–11.6)	0.006	2.27 (0.80–6.43)	0.124
Pathological stage III, yes	6.32 (3.40–11.8)	<0.001	4.25 (1.94–9.30)	<0.001
Serum CEA level ≥5.0 ng/mL	3.00 (1.77–5.08)	<0.001	1.59 (0.91–2.76)	0.101
Serum CA19‐9 level ≥ 37.0 U/mL	3.17 (1.70–5.90)	<0.001	1.39 (0.71–2.70)	0.333
Charlson Comorbidity Index, high	1.23 (0.72–2.09)	0.451		
Subcutaneous fat areas, low	1.36 (0.80–2.31)	0.250		
Visceral fat areas, low	2.21 (1.28–3.82)	0.005		
Fatty liver, yes	0.72 (0.39–1.32)	0.294		
Fatty replacement of pancreas, yes	0.81 (0.46–1.41)	0.453		
Skeletal mass index, low	2.16 (1.25–3.74)	0.006		
Body component score, low	2.45 (1.45–4.13)	<0.001	1.95 (1.13–3.35)	0.016

Abbreviations: CA19‐9, carbohydrate antigen 19–9; CEA, carcinoembryonic antigen; CI, confidence interval; HR, hazard ratio.

Table 3 presents the association between clinical variables and CSS. Univariate analysis found significant associations with CSS for BMI < 22 kg/m^2^ (*p* = 0.005), T3 or T4 tumors (*p* = 0.002), administrated adjuvant chemotherapy, (*p* = 0.003), R1 resection (*p* < 0.001), pathological stage III (*p* < 0.001) serum CEA level ≥5.0 ng/mL (*p* < 0.001), serum CA19‐9 level ≥ 37.0 U/mL (*p* < 0.001), low SFA (*p* = 0.011), low VFA (*p* < 0.001), low SMI (*p* = 0.034), and low BCS (*p* < 0.001). Multivariate analysis identified pathological stage III (*p* < 0.001; HR, 6.56; 95% CI, 2.48–17.3) and low BCS (*p* = 0.001; HR, 3.14; 95% CI, 1.57–6.27) as independent prognostic factors for CSS. A low BCS was associated with worse CSS (*p* < 0.001; log‐rank test for trend) (Figure 2C,D).

**TABLE 3:** Univariate and multivariate analyses of prognostic factors for cancer‐specific survival in patients with colorectal cancer after resection.

	Univariate analysis		Multivariate analysis	
Variables	HR (95% CI)	*p*‐value	HR (95% CI)	*p*‐value
Age ≥ 75 years	1.22 (0.66–2.26)	0.522		
Sex, male	1.78 (0.91–3.47)	0.091		
Body mass index <22 kg/m^2^	2.45 (1.31–4.59)	0.005	1.47 (0.72–2.98)	0.293
Right‐sided colon cancer, yes	0.89 (0.45–1.76)	0.733		
T3 or T4 tumor, yes	3.96 (1.67–9.41)	0.002	1.64 (0.62–4.32)	0.317
Adjuvant chemotherapy, yes	2.66 (1.41–5.04)	0.003	0.60 (0.25–1.44)	0.250
R1 resection, yes	6.11 (2.17–17.2)	<0.001	1.76 (0.57–5.40)	0.325
Pathological stage III, yes	6.13 (2.94–12.8)	<0.001	6.56 (2.48–17.3)	<0.001
Serum CEA level ≥5.0 ng/mL	3.31 (1.80–6.09)	<0.001	1.77 (0.91–3.40)	0.089
Serum CA19‐9 level ≥ 37.0 U/mL	4.04 (2.10–7.77)	<0.001	1.43 (0.66–3.10)	0.365
Charlson Comorbidity Index, high	1.79 (0.98–3.26)	0.059		
Subcutaneous fat areas, low	2.29 (1.21–4.33)	0.011		
Visceral fat areas, low	3.94 (1.94–8.00)	<0.001		
Fatty liver, yes	0.51 (0.24–1.10)	0.084		
Fatty replacement of pancreas, yes	0.60 (0.30–1.20)	0.144		
Skeletal mass index, low	1.98 (1.05–3.70)	0.034		
Body component score, low	4.36 (2.33–8.17)	<0.001	3.14 (1.57–6.27)	0.001

Abbreviations: CA19‐9, carbohydrate antigen 19–9; CEA, carcinoembryonic antigen; CI, confidence interval; HR, hazard ratio.

### 3.3 Subgroup analyses of BCS for disease‐free and cancer‐specific survival in patients with stage I–III CRC

**FIGURE 3:**
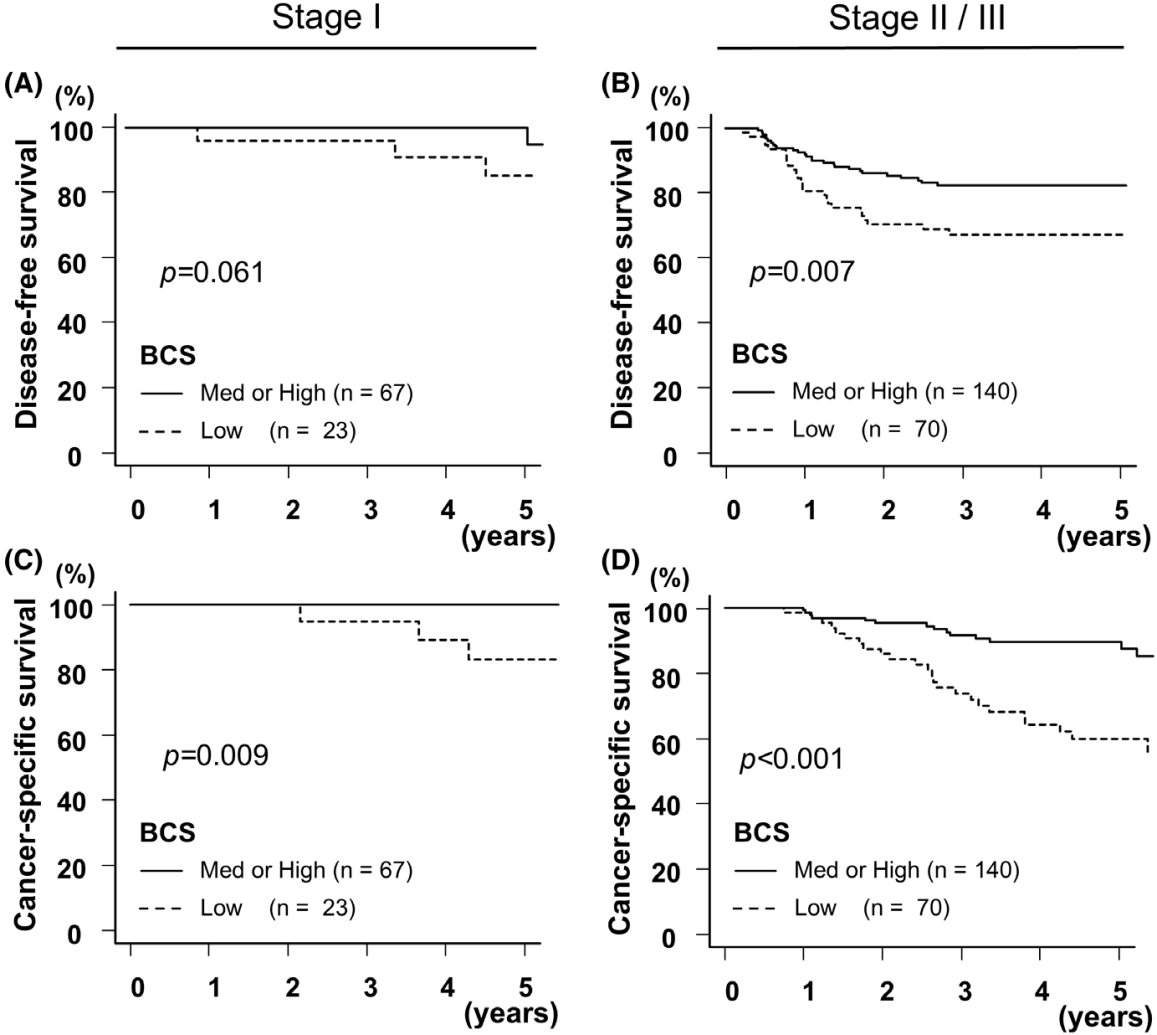
Kaplan–Meier curves for stage I or stage II/III cancer according to the status of body component score (BCS). Kaplan–Meier curves of disease‐free (A, B) and cancer‐specific survival (C, D) according to BCS status and cancer stage.

Subgroup analyses were performed for early or advanced stage in patients with stage I–III CRC according to the BCS. The Kaplan–Meier method evaluated the effect of a low BCS on early stage (stage I; *n* = 90) or advanced stage (stage II/III; *n* = 210). In early stage, patients with a low BCS showed a significantly reduced CSS (*p* = 0.009), but there were no significant differences in DFS (*p* = 0.061; Figure 3A,C). In advanced stage, patients with a low BCS exhibited a significantly decreased DFS (*p* = 0.007) and CSS (*p* < 0.001; Figure 3B,D). These results demonstrate that low BCS strongly affects survival in advanced CRC as well as in early stage of CRC.

## 4 DISCUSSION

In this study, we found that a low BCS was associated with poor DFS and CSS in patients with stage I–III CRC following radical resection. Recently, skeletal muscle, visceral fat, and subcutaneous fat have gained increasing attention as significant prognostic biomarkers.

Sarcopenia, the systemic loss of skeletal muscle due to aging, inactivity, poor nutritional, or malignancy‐related inflammation, is a known risk factor for postoperative complications and mortality in gastrointestinal cancers, including CRC.^14^, ^15^, ^16^ Obesity is a risk factor for perioperative complications^17^ and is typically evaluated using body mass index (BMI) as an indicator. Although BMI reflects to some extent body type (slender or obese), it does not provide a detailed representation of individual body component, because only height and weight are considered for its calculation. Body component indices from CT scans, such as SMI, SFA, and VFA, provide a more accurate representation of physical conditions and are related to CRC prognosis.^18^, ^19^ Studies have shown associations between body fat composition, such as the amount of SFA or VFA, and treatment outcomes in patients with malignant tumors, including CRC.^20^, ^21^ Ectopic fat accumulation in solid organs, particularly the liver, also affects CRC prognosis, with fatty liver associated with shorter survival in colorectal liver metastasis than normal liver; however, liver steatosis may prevent metastasis in the liver.^22^, ^23^ In addition, intra‐ and periorgan fat accumulations is interrelated with visceral and subcutaneous fat accumulation, independently of BMI.^24^ Skeletal muscle, body fat, and ectopic fat are closely related with each other, and have been reported to be individual risk factors for cancer survival. However, comprehensive body composition indices to evaluate these parameters are lacking. Therefore, we devised the BCS score.

Patients with a low BCS have low skeletal muscle and adipose fat, which may lead to significantly worse outcomes in our cohort attributed to interactive synergy between muscle and fat loss. In the subgroup analysis of our data, a low BCS was more associated with decreased survival in advanced CRC compared to early stage. These results might be explained by a synergistic effect between tumor growth and the cachexic environment resulting from low levels of skeletal muscle and the loss of adipose and ectopic fat. Malignant disease reduces muscle mass via decreased protein intake, endocrine signals, and increased inflammation.^22^ Low skeletal muscle promotes host systemic inflammation, characterized by a high neutrophil‐to‐lymphocyte ratio and low albumin levels, stimulating tumor cell proliferation and metastasis, worsening survival.^25^

In contrast, high adipose tissue levels provide a survival advantage for patients with cancer, as subcutaneous adipose is metabolically stable and produces leptin, improving insulin sensitivity. As insulin and insulin‐like growth factors cause tumor progression and metastasis, insulin sensitivity is crucial for preventing cancer cachexia and tumor recurrence.^26^ Moreover, Li et al. retrospectively analyzed 14 018 patients with cancer with low VFA and reported significantly worse survival, suggesting that VFA represents body fat mass and provides a potential indication of nutritional, immune, and inflammatory status.^27^ Furthermore, patients with visceral obesity may tolerate chemotherapy better than those with normal weight.^28^ Ectopic fat accumulation has also been identified as an important prognostic factor. Murono et al. reported that in patients with stage I–III CRC who underwent curative surgery, liver steatosis was associated with better liver‐specific DFS.^23^ Fat accumulation in the liver creates a favorable environment that prevents tumor cell invasion and metastasis.

Skeletal muscle and adipose tissues are not only structural components of the body but also endocrine organs, and this aspect is probably crucial for tumor growth in patients with a low BCS. Skeletal muscle synthesizes myokines such as myostatin, interleukin‐6, oncostatin M, and irisin. Exercise induces skeletal muscle to secrete myokines, which in turn suppress tumor cell development. Patients with low levels of skeletal muscle usually have decreased activity levels, leading to myokine imbalance. This is directly correlated with cancer progression, probably influencing proliferation, drug resistance, metabolic instability, and the epithelial‐mesenchymal transformation of cancer cells.^29^ In addition, some myokines are thought to regulate the tumor microenvironment in terms of angiogenesis or immune system activity.^29^ Although there are still many unknown aspects in the interaction of myokines and tumor cells, low levels of skeletal muscle and the resulting levels of myokines are possibly related to the formation of a favorable environment for tumor cells. Adipose tissues secrete adiponectin, with higher levels stimulating the production of proinflammatory cytokines, promoting colonic cell proliferation, and leading to an increase in the mortality risk of patients with CRC according to recent reports.^30^ Adequate levels of adipose tissue maintain host immunologic and metabolic stability, supporting nutritional capacity under anticancer systemic treatment stress.^28^ Also considering those sides of the endocrine organs of skeletal muscle and body fat cells, low BCS possibly reflects the disadvantage of prognosis for stage I–III CRC patients. Therefore, we hypothesized that low skeletal muscle, body weight, and ectopic fat create inflammatory, metabolic, and nutritional disadvantages, fostering cancer cell growth.

The BCS consists of three key elements: skeletal muscle, body fat, and ectopic fat, helping to identify high‐risk patients for strict follow‐up and possibly more aggressive adjuvant treatment. Long‐term interventions should be considered for patients with low BCS, especially for those with advanced tumors, to improve factors that are reversible (such as skeletal muscle or body fat), in parallel with adjuvant therapy. Our findings indicated the importance of evaluating both tumor factors and patient physical components to predict CRC long‐term outcomes.

This study had several limitations. First, the study design was retrospective and conducted at a single institution with a limited sample size. Second, we evaluated the VFA and SFA at the umbilicus level, which lacks a consensus measurement method. Definitions of fatty liver and pancreatic steatosis based on imaging are not fully established. Finally, only preoperative body components were assessed. Changes in body components caused by surgery or chemotherapy were not measured. A prospective randomized study or large, high‐quality cohorts should be involved to validate our findings.

## 5 CONCLUSION

This study demonstrated that a low BCS is significantly associated with DFS and CSS in patients with stage I–III CRC. This novel comprehensive body component index may provide a better prediction of the prognosis of patients with CRC.

## AUTHOR CONTRIBUTIONS

**Takashi Aida:** Data curation; formal analysis; visualization; writing – original draft; writing – review and editing. **Teppei Kamada:** Conceptualization; data curation; writing – review and editing. **Taigo Hata:** Project administration. **Junji Takahashi:** Project administration. **Kenei Furukawa:** Formal analysis. **Eisaku Ito:** Project administration. **Hironori Ohdaira:** Project administration; validation. **Masashi Yoshida:** Project administration. **Toru Ikegami:** Supervision. **Yutaka Suzuki:** Supervision.

## FUNDING INFORMATION

The authors received no specific funding for this work.

## CONFLICT OF INTEREST STATEMENT

Authors declare no conflict of interests for this article.

## ETHICS STATEMENT

Approval of the research protocol by an Institutional Reviewer Board: The study protocol was approved by the Institutional Review Board of the International University of Health and Welfare Hospital (21‐B‐22).

Informed Consent: The patients were given the opportunity to opt out of the study via public announcements.

Registry and the Registration No. of the study/trial: N/A.

Animal Studies: N/A.

## Supporting information


**Figure S1.** The proportion of patients with high SMI (A), SFA (B), VFA (C), Fat L (D) and Fat P (E) in each BCS group.
SMI, skeletal muscle index; SFA, subcutaneous fat area; VFA, visceral fat area;
Fat L, fatty liver; Fat P, pancreatic fatty replacement.
