# Supplementary material for: Novel body component score predicts long‐term survival in patients with stage I–III colorectal cancer following radical resection
Source: Ann Gastroenterol Surg. 2024 Nov 26;9(3):529–37. doi: 10.1002/ags3.12890 (PMC12080208; doi:10.1002/ags3.12890)
Supplement: Supplementary file 1 — Figure S1. The proportion of patients with high SMI (A), SFA (B), VFA (C), Fat L (D) and Fat P (E) in each BCS group. SMI, skeletal muscle index; SFA, subcutaneous fat area; VFA, visceral fat area; Fat L, fatty liver; Fat P, pancreatic fatty replacement. [file AGS3-9-529-s001.docx]

**Supplementary Fig. 1**


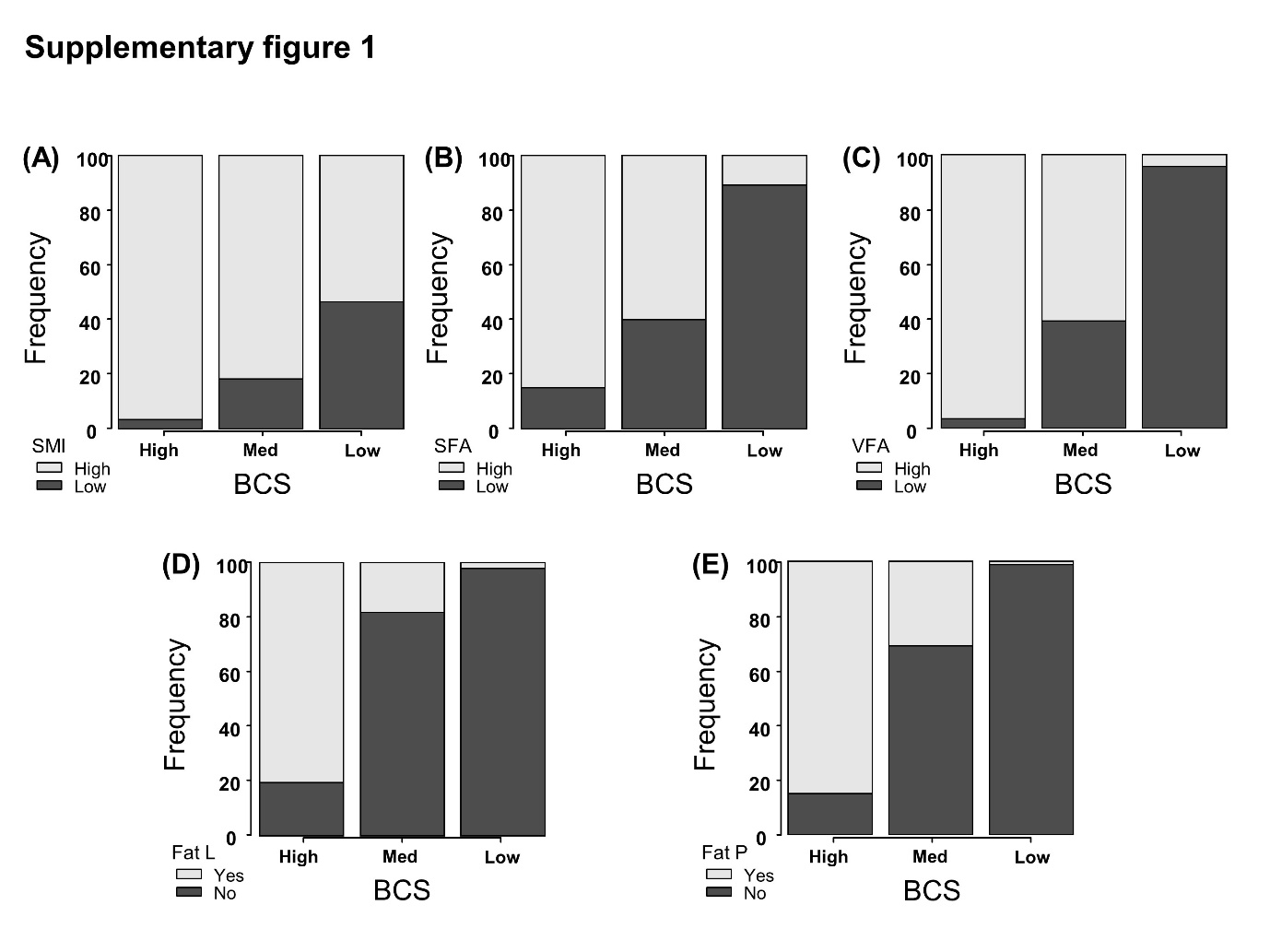


The proportion of patients with high SMI (A), SFA (B), VFA (C), Fat L (D) and Fat P (E) in each BCS group.

SMI, skeletal muscle index; SFA, subcutaneous fat area; VFA, visceral fat area;

Fat L, fatty liver; Fat P, pancreatic fatty replacement
